# Disease burden of infertility in five East Asian countries from 1990 to 2021 and prediction for 2050: An analysis of the Global Burden of Disease study 2021

**DOI:** 10.1371/journal.pone.0331617

**Published:** 2025-09-11

**Authors:** Fengze Sun, Wenyu Wang, Guixin Ding, Yini Wang, Hongquan Liu, Youwei Chi, Yicheng Guo, Xiaohong Ma, Jian Ma, Jitao Wu

**Affiliations:** 1 Department of Urology, Yantai Yuhuangding Hospital, Qingdao University, Yantai, Shandong, China; 2 The second clinical medical college, Binzhou Medical University, Yantai, Shandong, China; National Center for Chronic and Noncommunicable Disease Control and Prevention, Chinese Center for Disease Control and Prevention, CHINA

## Abstract

**Background:**

With the development of society, fertility seems to have become a public health issue in East Asian countries. At present, there is insufficient attention to infertility, especially male infertility. Understanding the burden and trends of infertility in men and women aged 20–49 years in East Asia is essential for fertility issues.

**Method:**

The related data in China, Democratic People’s Republic of Korea, Japan, Mongolia and Republic of Korea, was extracted from the Global Burden of Disease (GBD) Study 2021 including prevalence, disability-adjusted life-year (DALY), age-standardized prevalence rate (ASPR) and age-standardized DALY rate. Annual percentage change (APC) and average annual percentage change (AAPC) were conducted by joinpoint analysis. The impact of age, period and birth cohort on the infertility was calculated by age-period-cohort model. We also predicted the future burden of infertility using Bayesian age period cohort (BAPC) analysis.

**Result:**

The burden of infertility aged 20–49 years group varied across five East Asian countries from 1990 to 2021, in which the trend of China (AAPC: FI 0.12, 95%UI: 0.01 to 0.22; MI 0.14, 95%UI: 0.02 to 0.27) and Mongolia (AAPC: FI 0.3, 95%UI: 0.24 to 0.35; MI 0.25, 95%UI: 0.21 to 0.28) showed increased, and the others were stable. The risk of male prevalence was significantly higher than that of female in two developed countries, while it was contrary in three developing countries. The burden of infertility increased with age in developing countries and reached the highest at the age of 35–39. In developed countries, the 40–44 age group has the highest burden of infertility. The future burden of infertility showed a downward trend to 2050 in China and Democratic People’s Republic of Korea, while it predicted to increase in Japan, Mongolia and Republic of Korea. These differences may be affected by socio-economic factors and health policies, highlighting significant differences between countries.

**Conclusion:**

The infertility burden in the five East Asian countries aged 20–49 years varies among age group and gender from 1990 to 2021, which is still a public threat. It is necessary to formulate appropriate strategies according to different situations to alleviate the burden of infertility.

## Introduction

Clinical infertility is defined as the inability to conceive after 1 year of unprotected attempts and has emerged as a pressing public health priority. It is estimated that approximately 10–20% of couples worldwide suffer from infertility [1]. This condition not only affects the physical and mental health of individuals but also significantly diminishes their quality of life [2]. On a broader scale, infertility impacts society and the nation as a whole, contributing to population decline, increased pressure on social security systems, and constrained economic development [3].

Infertility can be categorized into female infertility (FI) and male infertility (MI), each with distinct causes and prevalence patterns. FI is often attributed to factors such as ovulation dysfunction, tubal blockage, endometriosis, uterine abnormalities, and cervical issues [4]. Several studies have leveraged the Global Burden of Disease (GBD) database to analyze the global prevalence and trends of infertility. FI tends to garner more attention due to prevailing social and traditional concepts. For instance, Shen et al. reported the trends of FI across global, regional, and national levels from 1990 to 2019 [5], while Liu et al. investigated the burden of endometriosis-related primary and secondary FI [6].

However, it is crucial to recognize that reproductive success depends on the combined contributions of both partners. MI is a significant contributor to infertility, accounting for 30%−50% of cases [7]. It is commonly associated with sexual dysfunction or poor semen quality [8–10]. Sun et al. described the global prevalence and disability-adjusted life-years (DALY) of MI using the GBD database from 2017 [11].

Previous studies have laid the foundation for a comprehensive global analysis of infertility. Infertility is not merely a medical issue concerning reproductive health; it also carries significant psychological, economic, and social implications. It can lead to trauma and stress, particularly in societies and cultures where fertility is highly emphasized [12]. In East Asia, for example, fertility is regarded as a vital social responsibility and family obligation. Consequently, infertility poses a substantial psychological burden, especially in the context of traditional beliefs and heightened societal expectations. Recent research indicates that the prevalence of female infertility is highest in East Asia [13].

In East Asian countries such as China, Japan, and the Republic of Korea, individuals over the age of 20 are considered adults. Therefore, this study focuses on the disease burden and trends of infertility among individuals aged 20–49 in East Asian countries, based on prevalence and DALY data. The objective of this study is to elucidate the trends and patterns of infertility burden, thereby providing critical insights for the effective management of infertility in East Asia.

## Method

### Data source

The data for this study was sourced from the Global Burden of Disease (GBD) 2021, an extensive database that compiles comprehensive information on 371 diseases and 88 risk factors across the globe, encompassing 5 Socio-Demographic Index (SDI) regions and 204 countries and territories [14]. In order to capture the widest array of information, multiple data types were included. For non-fatal information, data sources were collected from scientific literature, household survey data, epidemiological surveillance data, disease registry data, clinical informatics data, and other sources. For fatal information, data sources were mainly collected from vital registration and verbal autopsy, as well as survey, census, surveillance, cancer registry, police records, open-source databases, and minimally invasive tissue sampling [15]. Analyzing the data from GBD 2021 yields insights crucial for policymakers, health practitioners, and other key stakeholders. It enables a comprehensive understanding of disease occurrence and epidemic trends, thereby informing the development of effective health strategies.

The data on female and male infertility in China, the Democratic People’s Republic of Korea, Japan, Mongolia, and the Republic of Korea, including prevalence rates and disability-adjusted life years (DALY) estimates, can be accessed through the following link: https://vizhub.healthdata.org/gbd-results. This link is managed by the Institute for Health Metrics and Evaluation (IHME) at the University of Washington in the United States [14,15]. The cases of female infertility and male infertility were identified using the International Classification of Diseases, 10th revision (ICD-10) codes (N97-N98.9 for female infertility and N46-N46.02, N46.022-N46.12, N46.122-N46.9 for male infertility) and 9th revision (ICD-9) codes (628–628.9, V26-V26.49, V26.51, V26.8-V26.9, V59.7-V59.74 for female infertility and 606–606.9, V26.5, V26.52 for male infertility).

### Ethics statement

The dataset employed in this investigation was sourced exclusively from the GBD 2021 database which was conducted in accordance with the ethical guidelines established by the Declaration of Helsinki and complies with relevant jurisdictional regulatory requirements. As this secondary analysis exclusively utilized publicly accessible aggregated data from an open-source epidemiological database, it was exempted from requiring ethics committee approval under applicable institutional guidelines.

### Joinpoint regression analysis

Joinpoint regression analysis was conducted to evaluate temporal trends in East Asia’s infertility burden (1990–2021), identifying significant trend changes (joinpoints) that divide the overall period into distinct phases. Annual percentage change (APC) quantified annual variations within each phase, while average APC (AAPC) characterized cumulative trends. Upward trends required both estimated AAPC and its 95% CI lower limit >0; downward trends necessitated AAPC and 95% CI upper limit <0. This segmentation enabled clearer interpretation of burden fluctuations across chronological intervals.

### Age-period-cohort analysis

The APC model was used to evaluate the effects of age, period and cohort in disease burden of infertility. Age effect analysis represented the impact of different age group on outcomes; period effect analysis evaluated disease risk across different years; cohort effect analysis examined risk changes between different groups with same birth years. In this study, we divided 20–49 years old into six age groups according to five years. Because 1990–1991 was less than 5 years, 1992–2021 was divided into six period groups according to five years to involve period effect analysis. The APC model was helpful to understand the disease burden of different groups of people, and was available on the National Cancer Institute (NCI) website (https://analysistools.cancer.gov/apc/) [16].

### Statistical analysis

In this study, we utilized prevalence and DALYs as metrics to assess the global burden of female and male infertility. DALYs are computed by combining years lived with disability (YLDs) and years of life lost (YLLs), reflecting the unhealthy lost survival time attributed to disease [17]. In order to make the data from different regions comparable, these indicators were qualified by using age-standardized rate (ASR). The ASR was computed per 100,000 persons utilizing the following formula: $ASR=\frac{\Sigma_{i=1}^{A}a_{i}w_{i}}{\Sigma_{i=1}^{A}w_{i}}\times 100,000$ ($a_{i}$:the rate in i^th^ age group;$w_{i}$: the number of people in the same i^th^ age group among the standard population; A: the number of age groups).

We also adopted Bayesian age-period-cohort (BAPC) model comprising integrated nested Laplace approximations to predict the future trend of infertility burden. Compared with other prediction method, the BAPC method had better accuracy by previous studies [18,19].

All analytical procedures and visual representation were performed by using R software (version 4.3.1) with using R packages including “ggplot2”, “BAPC”, “dplyr”, “RcolorBrewer”, “patchwork”.

## Result

### National level description

**Fig 1:**
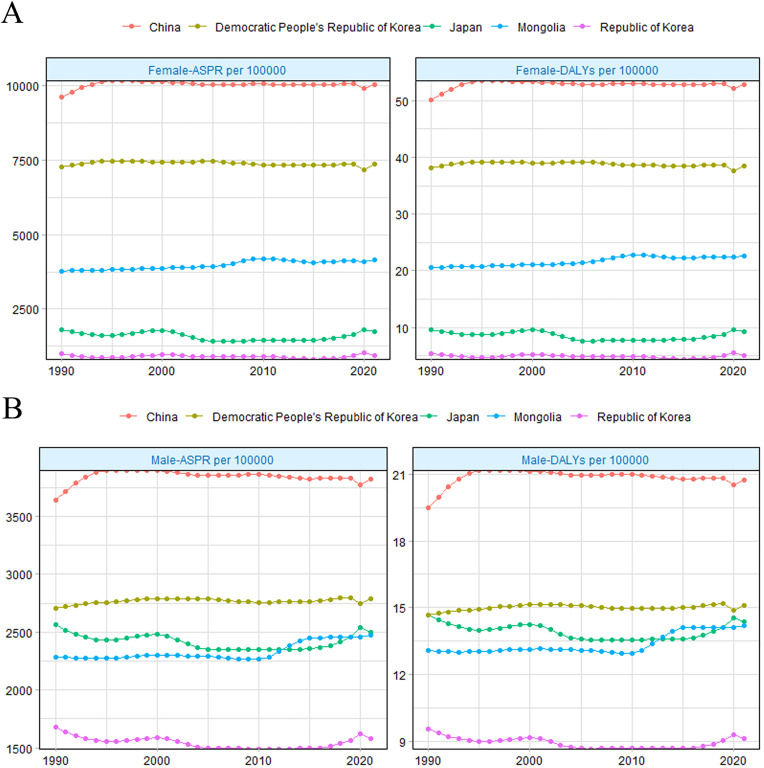
National trend of infertility burden. Trends of infertility burden of ASPR and aged-standardized DALY rate in female (A) and male (B) aged 20-49 years among five East Asian countries from 1990 to 2021.

Between 1990 and 2021, the prevalence of FI and MI among individuals aged 20–49 years exhibited divergent trends across East Asian countries. Specifically, the prevalence cases of FI and MI increased in China, the Democratic People’s Republic of Korea, Mongolia and Republic of Korea, while it decreased in Japan (Table 1). The trends in DALYs mirrored those of prevalence, suggesting that changes in prevalence were a dominant factor influencing DALYs (Fig 1).

**Table 1:** Prevalence and DALYs of female and male infertility between 1990 and 2021.

Sex Burden	Location	1990		2021		AAPC (95% UI)
		Number (95% UI)	ASR (95% UI)	Number (95% UI)	ASR (95% UI)	
**Female**						
**Prevalence**						
	China	24696219 (7108865,55867146)	9613.38 (2752.95,21727)	29289260 (7995885,67430685)	10043.19 (2745.91,23164.75)	0.12 (0.01, 0.22)
	Democratic People’s Republic of Korea	313051 (80770,746703)	7272.93 (1891.98,17270.48)	410154 (106880,974464)	7356.22 (1913.78,17453.64)	0.00 (−0.16, 0.17)
	Japan	558301 (27664,2174917)	1789.77 (94.07,7282.37)	424183 (22334,1674068)	1746.18 (97.25,7159.59)	0.06 (−0.30, 0.41)
	Mongolia	15622 (2902,40891)	3781.13 (620.41,10150.66)	30942 (4778,83260)	4164.18 (672.34,11109.63)	0.30 (0.24, 0.35)
	Republic of Korea	91549 (5636,437723)	991.67 (57.95,4555.35)	112092 (6774,526328)	950.98 (60.58,4662.36)	0.07 (−0.57, 0.72)
**DALYs**						
	China	128898 (28372,373555)	50.04 (11,145.24)	153093 (32139,452298)	52.75 (10.98,156.16)	0.15 (0.05, 0.25)
	Democratic People’s Republic of Korea	1647 (322,4893)	38.1 (7.52,112.77)	2146 (427,6382)	38.51 (7.66,114.34)	0.00 (−0.22, 0.22)
	Japan	2979 (110,13897)	9.59 (0.37,45.86)	2257 (89,10954)	9.32 (0.39,46.21)	0.04 (−0.30, 0.38)
	Mongolia	86 (12,266)	20.54 (2.46,65.87)	167 (20,537)	22.67 (2.8,72.21)	0.30 (0.25, 0.35)
	Republic of Korea	499 (19,2744)	5.38 (0.19,28.78)	602 (24,3234)	5.13 (0.21,28.28)	0.04 (−0.55, 0.64)
**Male**						
**Prevalence**						
	China	10127176 (3493950,21871167)	3642.84 (1252.27,7850.17)	11761437 (4174957,25339229)	3821.12 (1365.76,8245.33)	0.14 (0.02, 0.27)
	Democratic People’s Republic of Korea	111419 (39065,254206)	2708.02 (953.55,6160.37)	176994 (60667,396054)	2788.49 (955.99,6236.96)	0.07 (−0.08, 0.23)
	Japan	741648 (252503,1683820)	2561.5 (870.25,5800.15)	551353 (188539,1232593)	2501.2 (847.61,5585.06)	−0.03 (−0.16, 0.10)
	Mongolia	9277 (3580,20523)	2282.44 (870.11,5060.45)	18239 (6584,41661)	2470.78 (893.78,5617.44)	0.25 (0.21, 0.28)
	Republic of Korea	175414 (58969,403685)	1676.02 (563.26,3855.73)	221058 (76733,515229)	1580.35 (545.6,3746.36)	−0.13 (−0.29, 0.03)
**DALYs**						
	China	54363 (13687,150360)	19.48 (4.91,53.91)	63405 (16611,175166)	20.75 (5.41,57.06)	0.19 (0.07, 0.31)
	Democratic People’s Republic of Korea	606 (150,1733)	14.69 (3.67,41.99)	956 (239,2694)	15.1 (3.76,42.53)	0.07 (−0.05, 0.19)
	Japan	4194 (1000,11839)	14.68 (3.5,41.2)	3257 (804,9071)	14.36 (3.55,39.76)	−0.03 (−0.15, 0.08)
	Mongolia	54 (14,141)	13.07 (3.45,34.64)	104 (26,284)	14.19 (3.61,38.6)	0.26 (0.23, 0.29)
	Republic of Korea	1013 (239,2865)	9.58 (2.26,27.16)	1073 (257,2989)	9.13 (2.19,25.42)	−0.10 (−0.29, 0.09)

For FI, the ASPR and age-standardized DALY rate followed a consistent ranking from highest to lowest: China, the Democratic People’s Republic of Korea, Mongolia, Japan, and the Republic of Korea (Fig 1A). In Mongolia, the ASPR of FI increased from 3,781.13 cases per 100,000 persons in 1990–4,164.18 cases per 100,000 persons in 2021, with an AAPC of 0.3 (95%UI: 0.24 to 0.35) (Table 1). A similar upward trend was observed in China, with an AAPC of 0.12 (95% UI: 0.01 to 0.22). In contrast, the Democratic People’s Republic of Korea, Japan, and the Republic of Korea maintained stable infertility burdens, with AAPCs of 0.0 (95% UI: −0.16 to 0.17), 0.06 (95% UI: −0.3 to 0.41), and 0.07 (95% UI: −0.57 to 0.72), respectively (Table 1).

The trends for MI were comparable (Table 1, Fig 1B). China had the highest ASPR for MI among the five countries, with an increasing trend (AAPC: 0.14, 95% UI: 0.02 to 0.27). The Republic of Korea had the lowest ASPR and maintained a stable trend (AAPC: −0.13, 95% UI: −0.29 to 0.03) from 1990 to 2021. The Democratic People’s Republic of Korea also exhibited a stable MI burden (AAPC: 0.07, 95% UI: −0.28 to 0.03). Notably, Japan’s ASPR for MI surpassed that of Mongolia before 2012, but this pattern reversed after that (Fig 1B). Japan’s MI trend remained stable (AAPC: −0.03, 95% UI: −0.16 to 0.1), while Mongolia experienced an increase (AAPC: 0.25, 95% UI: 0.21 to 0.28) (Table 1).

### Sex and age patterns

**Fig 2:**
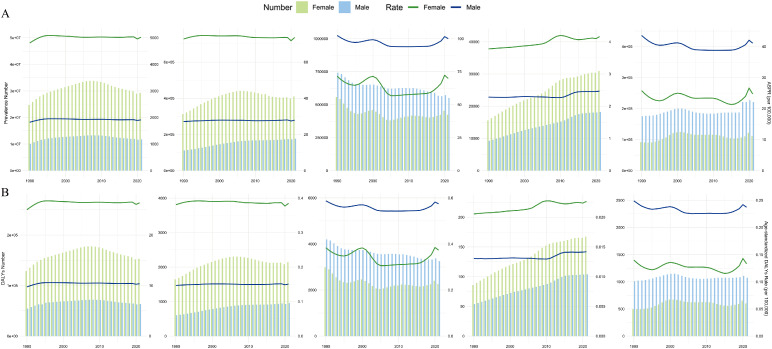
Age and national distribution of prevalence and DALYs. The number and ASR of prevalence (A) and DALYs (B) by sex in five East Asian countries from 1990 to 2021.

In the five East Asian countries—China, the Democratic People’s Republic of Korea, Japan, Mongolia, and the Republic of Korea—infertility exhibited distinct patterns in terms of prevalence and DALYs across different age groups and countries. In 2021, China, the Democratic People’s Republic of Korea, and Mongolia showed a similar disease burden, with the number of FI cases far exceeding MI cases in each age group, except for the 20–24 years age group (S1-S2 Figs in S1 File). Conversely, in Japan and the Republic of Korea, MI cases outnumbered FI cases in all age groups except the 40–44 years age group. From 1990 to 2021, the trends of FI and MI were consistent within each country (Fig 2A, 2B).

To better observe these trends, we divided the 20–49 years age range into six groups. In 2021, China had the highest ASR of FI across all age groups (S3A Fig in S1 File). For MI, individuals aged 25–44 years in China had the highest ASR, while Mongolia had the highest ASR in the 20–24 years age group, and Japan had the highest ASR in the 45–49 years age group (S3B Fig in S1 File). We further evaluated the trends in each age group from 1990 to 2021 across different countries (S4 Fig in S1 File). China consistently had the highest ASPR and age-standardized DALY rate for FI across all six age groups during this period (S4A Fig in S1 File). For MI, China had the highest ASR among individuals aged 25–44 years from 1990 to 2021 (S4B Fig in S1 File). However, in the 20–24 years age group, China had a higher ASPR from 1995 to 2012, while Mongolia had a higher ASPR from 2013 to 2021. Japan had the highest ASR for MI in the 45–49 years age group throughout the study period. We also assessed the trends of different age groups within each country (S5 Fig in S1 File). Compared to other age groups, individuals aged 35–39 years had the highest ASR of infertility in China, the Democratic People’s Republic of Korea, and Mongolia from 1990 to 2021 (S5A, S5B, S5D Fig in S1 File). In contrast, individuals aged 40–44 years had the highest ASR in Japan and the Republic of Korea (S5C, S5E Fig in S1 File). Notably, the 45–49 years age group had the lowest ASR across all five countries from 1990 to 2021.

### Joinpoint analysis

**Fig 3:**
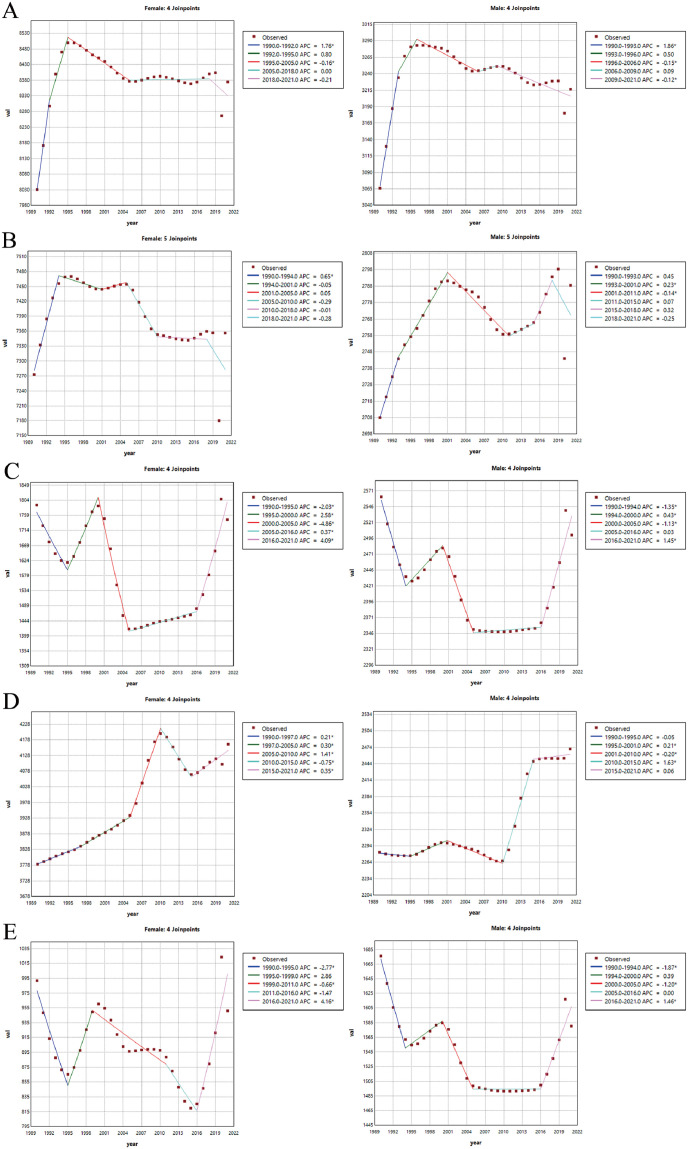
Joinpoint regression analysis. Joinpoint regression analysis of the APC in the prevalence of female and male infertility diseases from 1990 to 2021 across five East Asian countries including China **(A)**, Democratic People’s Republic of Korea **(B)**, Japan **(C)**, Mongolia **(D)**, and Republic of Korea **(E)**.

In the joinpoint analysis of infertility trends across the five East Asian countries, the prevalence rates exhibited complex and dynamic patterns from 1990 to 2021 (Fig 3, S1 Table). In China, although the overall trends for both FI and MI were upward, the increases were concentrated in specific periods. For FI, the significant rise occurred from 1990 to 1995, with APC of 1.76 (95% CI: 0.89 to 2.64) from 1990 to 1992 and 0.8 (95% CI: −0.05 to 1.66) from 1992 to 1995. For MI, the most notable increase was from 1990 to 1996, with APC values of 1.86 (95% CI: 1.4 to 2.31) from 1990 to 1993 and 0.5 (95% CI: −0.35 to 1.40) from 1993 to 1996. Following these periods, both FI and MI showed a gradual decline (Fig 3A). In the Democratic People’s Republic of Korea, infertility burden increased and remained at a higher level throughout the 1990s, but has shown a downward trend in the past four years (Fig 3B). Japan and the Republic of Korea exhibited similar patterns of infertility burden, characterized by significant fluctuations from 1990 to 2021 (Fig 3C, 3E). Initially, both FI and MI prevalence decreased, followed by periods of both increases and decreases in the middle years. However, since 2016, there has been a notable upward trend in the prevalence of both FI and MI in both countries. In Mongolia, the overall trends for FI and MI were significantly upward. The most substantial growth in FI occurred from 2005 to 2010, with an APC of 1.41 (95% CI: 1.21 to 1.60), while the most significant increase in MI was from 2010 to 2015, with an APC of 1.63 (95% CI: 1.51 to 1.75) (Fig 3D, S1 Table).

These findings highlight that the prevalence of infertility in different countries has undergone dynamic and complex changes over various time periods. This underscores the necessity for developing targeted public health strategies to effectively address these burdens.

### Age-period-cohort analysis

#### Longitudinal age curve.

**Fig 4:**
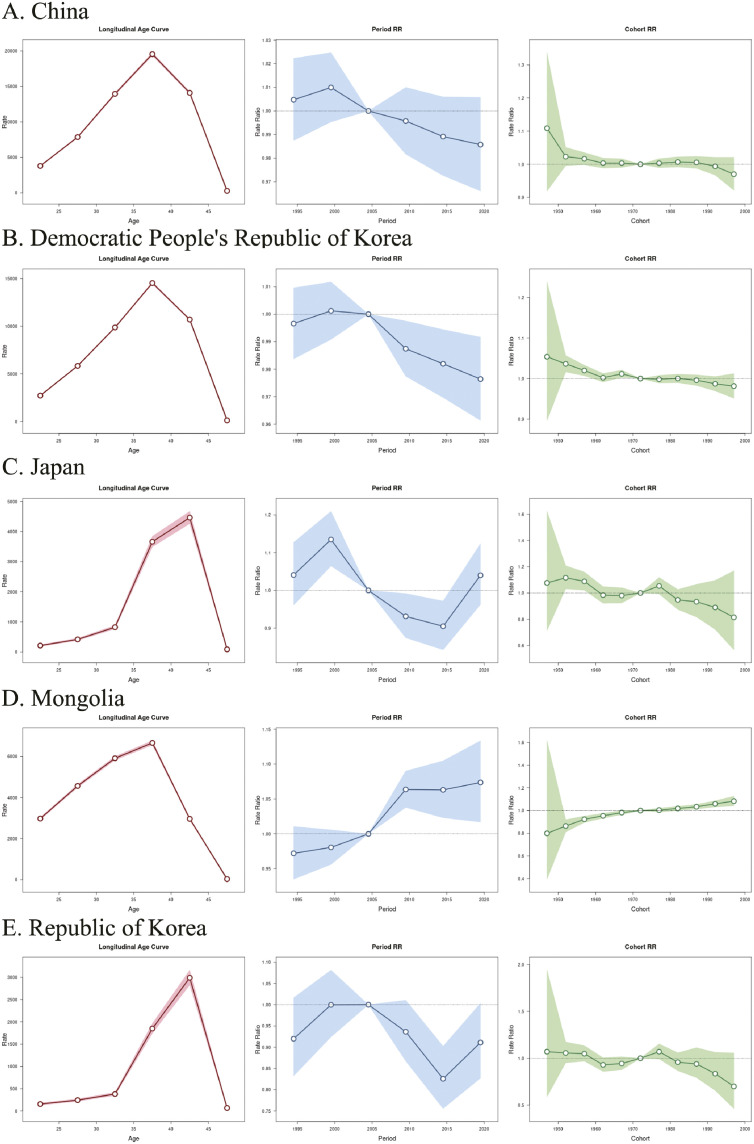
Age-period-cohort analysis in female. Age-period–cohort effects model depicting the burden of female infertility across five East Asian countries including China **(A)**, Democratic People’s Republic of Korea **(B)**, Japan **(C)**, Mongolia **(D)**, and Republic of Korea **(E)**.

The prevalence of infertility significantly increases with age and then decreases across five East Asian countries (Fig 4, S6 Fig in S1 File). Differently, the highest risk of FI occurring in the 35–39 age group across three developing countries including China, Democratic People’s Republic of Korea and Mongolia, while 40–44 age group was observed in developed countries including Japan and Republic of Korea. In MI, the longitudinal age curves of MI were similar to FI.

#### Period effect.

The analysis of the period effect revealed that the relative risk of infertility disease varied among the five East Asian countries at different times from 1992 to 2021 (Fig 4, S6 Fig in S1 File). In Mongolia, a continuous upward trend was observed in FI and MI. China experienced growth in the early stages, but gradually declined afterwards. It was seen as a growth trend at recent period in Democratic People’s Republic of Korea, Japan and the Republic of Korea.

#### Cohort effect.

Cohort effect analyses showed substantial differences in the risk of developing infertility diseases across different birth years in five East Asian countries (Fig 4, S6 Fig in S1 File). In Mongolia, disease risk continued to increase as birth year increased. In the other four countries, the risk of infertility was lower in late birth cohorts, which indicated that lower disease risk was seen among younger generations.

### Future forecasts

Based on the GBD 2021 data, we projected the future disease burden of infertility in five East Asian countries through to 2050 (S7 Fig in S1 File). Our analysis indicates that China and the Democratic People’s Republic of Korea will experience a declining trend in the ASPR of infertility for both females and males by 2050 (S7A, S7B Fig in S1 File). Conversely, Japan, Mongolia, and the Republic of Korea are projected to see a more pronounced increase in ASPR by 2050 (S7C, S7D, S7E Fig in S1 File). Specifically, for females, the ASPR in Japan is projected to rise to 2,726.68 cases per 100,000 by 2050. In Mongolia, the rate is expected to reach 5,055.3 cases per 100,000, while the Republic of Korea will see an increase to 1,239.31 cases per 100,000. For males, the ASPR in Japan will climb to 3,420.29 cases per 100,000 by 2050. Mongolia is projected to reach 2,681.3 cases per 100,000, and the Republic of Korea will see an increase to 2,120.3 cases per 100,000. Governments in Japan, Mongolia, and the Republic of Korea should consider implementing measures to address the anticipated rise in infertility cases, thereby mitigating the potential impact on individuals and society.

## Discussion

Globally, the fertility rate in most countries and regions has been gradually declining, while the prevalence of infertility has been steadily increasing [3]. This demographic shift poses a significant threat to population structures and has far-reaching implications for the economic and social development of countries and regions worldwide. Although many countries have recognized this challenge and implemented measures to address it, the outcomes have been less than satisfactory. It is essential to fully understand the unique circumstances of each country and region in order to develop tailored solutions that optimize resource allocation and effectively tackle these issues. The latest research highlights that, despite the overall upward trend in infertility prevalence, substantial variations persist across regions, genders, and age groups [13]. Specifically, East Asia has the highest prevalence of female infertility globally, while its male infertility prevalence ranks third. Additionally, the 15–19 age group exhibits a lower prevalence rate, which is unsurprising given that individuals in this age range are still considered minors in many East Asian countries. Therefore, a comprehensive understanding of the infertility burden among individuals aged 20–49 in East Asia is crucial.

This study is the first to investigate the trends and burden of infertility among individuals aged 20–49 in five East Asian countries from 1990 to 2021, using data from GBD 2021 database. The burden estimates for FI and MI varied significantly across countries during the observational period, as measured by prevalence and DALYs. These trends and burdens are influenced by a multitude of factors, including cultural, economic, and policy-related aspects [20]. We believe that this study will draw greater attention from public health experts to the issue of infertility and provide valuable insights to help mitigate this growing public health challenge.

Overall, developing countries in East Asia, such as China, the Democratic People’s Republic of Korea, and Mongolia, tend to have a higher burden of infertility, while developed countries like Japan and South Korea exhibit lower prevalence rates. In high-income countries, the healthcare system is generally more advanced, providing better access to medical assistance, which can help mitigate infertility-related challenges [21,22]. In contrast, developing countries still face significant social pressures as they strive to improve their healthcare infrastructure. Some studies have demonstrated that increased stress in the workplace can contribute to higher infertility rates [23,24]. This is particularly evident in certain high-stress occupations. For example, medical professionals, especially surgeons, have been shown to experience higher rates of miscarriage, infertility, and pregnancy complications [25]. Infertility not only affects their personal lives, causing anxiety and other emotional distress, but also creates a vicious cycle that further exacerbates these issues [26]. This pattern is particularly pronounced in FI, which has shown significant disparities across countries from 1990 to 2021. Before 2013, Japan had a higher burden of MI compared to Mongolia. However, with economic development, the incidence of MI in Mongolia has gradually increased, reflecting an overall upward trend in infertility burden from 1990 to 2021. This trend may be related to economic growth and increased awareness of infertility issues. Given the limited research on infertility in Mongolia, our findings may help fill gaps in understanding. Considering the imbalance of economic development on infertility, developing countries should invest more medical resources including reducing examination and treatment costs, as well as free mental health counseling.

Our analysis also revealed that the burden of infertility among women in developing countries is significantly higher than that among men, while in developed countries, the prevalence of infertility in men exceeds that in women. Reproductive health is particularly crucial for female fertility, as any related issues—such as ovarian cysts, endometriosis, or fallopian tube obstruction—can lead to infertility [27]. In developed countries, robust healthcare systems ensure that women can promptly identify and address these problems. In contrast, developed countries like Japan and the Republic of Korea are characterized by high work pressure, with men often shouldering more work responsibilities. Long hours of high-intensity work result in physical fatigue and mental stress, which can disrupt the endocrine system and negatively impact sperm production and quality [28]. Additionally, the widespread adoption of male health check-ups in these countries leads to a higher detection rate of male infertility [29]. Increasing attention is now being paid to male infertility, with studies indicating that male factors contribute to over 50% of infertile couples [30]. In developing countries, however, social and economic perceptions of infertility differ. In some regions, women may face greater societal pressure regarding marriage and childbirth, leading to higher rates of medical examination and treatment among women. This, in turn, results in a more pronounced detection of female infertility. Conversely, infertility in men often has less social impact, and approximately 25% of men in infertile couples remain untested, potentially underestimating the true prevalence of male infertility [31,32]. Therefore, infertility education should be expanded and both partners should be encouraged to undergo testing and seek solutions together, especially in developing countries. Strengthening social care for men is also necessary in developed countries.

Between 1990 and 2021, the prevalence of infertility among women and men aged 20–49 increased with age in developing countries, including China, the Democratic People’s Republic of Korea, and Mongolia, peaking in the 35–39 age group. In contrast, developed countries such as the Republic of Korea and Japan saw the highest infertility prevalence in the 40–44 age group. These trends may be related to the reproductive needs and fertility patterns of different age groups. In modern society, as educational levels have risen and career aspirations have gained greater importance, the average age of marriage has been postponed, leading to a gradual increase in the age of first childbirth [33]. For instance, a survey in the United States revealed that the average age of first-time mothers increased from 24.9 years in 2000 to 26.3 years in 2014 [34]. Research has consistently shown that fertility declines progressively with age, with a more rapid decline observed after the age of 30 [35]. In developed countries, the issue of population aging is particularly pronounced, resulting in a higher proportion of older couples seeking to have children [36]. These couples are more likely to seek medical attention and undergo relevant examinations, leading to higher reported rates of infertility. Conversely, at the end of the childbearing years, couples’ fertility needs and reproductive capacity are significantly lower compared to other age groups. As a result, infertility is less of a concern for this group, and the proportion of individuals aged 45–49 reporting infertility is the lowest. Due to the continuous postponement of childbearing age, it is necessary to strengthen the implementation of social welfare, reduce their economic burden, and conduct early infertility test, especially for young people. Considering the trend of aging population, the government should strengthen medical assistance for elder couples with infertility.

In our joinpoint analysis, we observed that the burden of infertility in East Asian countries is highly dynamic and may be significantly influenced by economic, social, and policy factors. Since China implemented stricter family planning policies in 1995, the prevalence of infertility significantly decreased with decline in fertility rates. The fertility demand of the Japanese population increased significantly with the implementation of the fertility promotion policies in 1995, 2003 and 2016, leading to a noticeable increase in the detection rate of infertility among elder couples [37]. Our results also found that the burden of infertility in South Korea significantly increased based on the aging population since the implementation of comprehensive fertility promotion policies in 2016. Based on the current cohort, our prediction analysis projects that by 2050, China and the Democratic People’s Republic of Korea will continue to see a decline in infertility burden, whereas Japan, Mongolia, and the Republic of Korea are expected to face an upward trend. This underscores the need for these countries to prioritize infertility issues and consider further increasing investment in polices. For Mongolia, in particular, it is crucial for the government to adopt healthcare strategies that align with economic growth. By doing so, they can better protect public health and promote sustainable economic and cultural development.

Although infertility will not seriously affect the quality of life and level of individuals like cancer, it is of great significance for future social, economic and demographic development. Infertility may also increase the risk of cancer, both in men and women [38]. Infertility is caused by 85% of infertile couples’ abnormal physiological functions or potential diseases. The most common causes of infertility are ovulation dysfunction, male infertility and oviduct disease. The remaining 15% of infertile couples suffer from “unexplained infertility” [39]. Infertility caused by related diseases should be treated actively. For example, infertility caused by female fallopian tube obstruction can be treated surgically. Infertility caused by male erectile dysfunction can be treated by medication or surgery. For unexplained infertility, assisted reproductive technology can now be used to help infertile couples with reproductive needs, including in vitro fertilization, in vitro fertilization embryo transfer [40]. These technologies are worth promoting widely as effective in alleviating the burden of infertility, especially in developing countries. At the same time, some unhealthy lifestyles and diets can also lead to infertility [39,41]. For infertile couples, it is recommended to actively seek pre pregnancy lifestyle counseling to improve the chances of live birth.

This study investigated the general distribution and trends of infertility in couples aged 20–49 years in East Asian countries from 1990 to 2021. We have made a detailed analysis according to different ages and genders in different countries to understand the differences. Our results will help to allocate resources and plan health services for more infertile patients. But our research also has some limitations. First, differences in disease reporting systems and data collection methods among countries may affect the accuracy of the results, especially in the Democratic People’s Republic of Korea and Mongolia. Secondly, cultural differences and economic level affected people’s understanding and statistics of male infertility. This might also be the reason for the relatively low incidence of male infertility. The whole society needs to further strengthen the popularization of infertility related knowledge. Thirdly, the lack of risk factors affecting infertility in the database made us unable to fully understand the degree of each risk factor and fully allocate resources. Finally, our prediction model for the future incidence is based on the trend given by the current data, and does not include the future regional policies, medical technology progress and other factors, which may have a deviation on the accuracy of the prediction.

In short, from the current data, we concluded that there were great differences in the burden of infertility in different countries, which had a certain impact on the future socio-economic development. We have put forward some suggestions regarding the burden of different countries. This study provides some data support for the formulation of these policies, which can make the allocation of resources more reasonable. More research is also needed to clarify the impact of various risk factors on the burden of infertility, so that resources can be more accurately allocated.

## Conclusion

The results of this study show that the infertility burden of the five East Asian countries aged 20–49 years is different among different countries from 1990 to 2021, which is still a public threat. The burden of infertility in developing countries is significantly higher than that in developed countries. In developed countries, the burden of MI is significantly higher than that of FI, and the highest age of onset is 40–44 years old. In developing countries, the burden of FI is heavier, mostly at the age of 35–39. The burden of infertility may have a significant impact on social and economic development. Each country should formulate effective public health intervention measures according to the actual situation to alleviate the current situation.

## Supporting information

S1 File S1 Fig. Age and sexual distribution of ASPR in 2021. ASPR of infertility diseases in five East Asian countries in 2021, broken down by age group and sex. S2 Fig. Age and sexual distribution of aged-standardized DALY rate in 2021. Aged-standardized DALY rate of infertility diseases in five East Asian countries in 2021, broken down by age group and sex. S3 Fig. Age and national burden in 2021. The ASR of prevalence and DALYs grouped by age group among five East Asian countries in 2021 between female (A) and male (B) infertility burden. S4 Fig. National trend among different age group. Trends of female (A) and male (B) infertility burden of ASPR and aged-standardized DALY rate among five East Asian countries from 1990 to 2021, grouped by 20-24 year group, 25-29 year group, 30-34 year group, 35-39 year group, 40-44 year group, 45-49 year group. S5 Fig. Trend of age burden among five countries. Trends of female and male infertility burden of ASPR and aged-standardized DALY rate grouped by age group from 1990 to 2021 among China (A), Democratic People's Republic of Korea (B), Japan (C), Mongolia (D), and Republic of Korea (E). S6 Fig. Age-period-cohort analysis in male. Age-period–cohort effects model depicting the burden of male infertility across five East Asian countries including China (A), Democratic People's Republic of Korea (B), Japan (C), Mongolia (D), and Republic of Korea (E). S7 Fig. The future forecasts to 2050. The forecast of infertility to 2050 in ASPR among China (A), Democratic People's Republic of Korea (B), Japan (C), Mongolia (D), and Republic of Korea (E).
(ZIP)

S1 Table Joinpoint regression analysis: trends in age-standardized prevalence of female infertility and male infertility among five East Asian countries from 1990 to 2021. (DOCX)
