# Supplementary material for: Disease burden of infertility in five East Asian countries from 1990 to 2021 and prediction for 2050: An analysis of the Global Burden of Disease study 2021
Source: PLoS One. 2025 Sep 11;20(9):e0331617. doi: 10.1371/journal.pone.0331617 (PMC12425187; doi:10.1371/journal.pone.0331617)
Supplement: S1 File — ASPR of infertility diseases in five East Asian countries in 2021, broken down by age group and sex. S2 Fig. Age and sexual distribution of aged-standardized DALY rate in 2021. Aged-standardized DALY rate of infertility diseases in five East Asian countries in 2021, broken down by age group and sex. S3 Fig. Age and national burden in 2021. The ASR of prevalence and DALYs grouped by age group among five East Asian countries in 2021 between female (A) and male (B) infertility burden. S4 Fig. National trend among different age group. Trends of female (A) and male (B) infertility burden of ASPR and aged-standardized DALY rate among five East Asian countries from 1990 to 2021, grouped by 20-24 year group, 25-29 year group, 30-34 year group, 35-39 year group, 40-44 year group, 45-49 year group. S5 Fig. Trend of age burden among five countries. Trends of female and male infertility burden of ASPR and aged-standardized DALY rate grouped by age group from 1990 to 2021 among China (A), Democratic People's Republic of Korea (B), Japan (C), Mongolia (D), and Republic of Korea (E). S6 Fig. Age-period-cohort analysis in male. Age-period–cohort effects model depicting the burden of male infertility across five East Asian countries including China (A), Democratic People's Republic of Korea (B), Japan (C), Mongolia (D), and Republic of Korea (E). S7 Fig. The future forecasts to 2050. The forecast of infertility to 2050 in ASPR among China (A), Democratic People's Republic of Korea (B), Japan (C), Mongolia (D), and Republic of Korea (E). (ZIP) [file pone.0331617.s001.zip › S1 Table.docx]

**S1 Table.** Joinpoint regression analysis: trends in age-standardized prevalence of female infertility and male infertility among five East Asian countries from 1990 to 2021.

| **Location** | **Female** | | **Male** | |
| --- | --- | --- | --- | --- |
|  | **Period** | **APC**  **(95% UI)** | **Period** | **APC**  **(95% UI)** |
| **China** | 1990-1992 | 1.76  (0.89, 2.64) | 1990-1993 | 1.86  (1.40, 2.31) |
|  | 1992-1995 | 0.80  (-0.05, 1.66) | 1993-1996 | 0.50  (-0.39, 1.40) |
|  | 1995-2005 | -0.16  (-0.24, -0.08) | 1996-2006 | -0.15  (-0.23, -0.07) |
|  | 2005-2018 | 0.00  (-0.05, 0.05) | 2006-2009 | 0.09  (-0.80, 0.99) |
|  | 2018-2021 | -0.21  (-0.65, 0.22) | 2009-2021 | -0.12  (-0.17, -0.07) |
| **Democratic People's Republic of Korea** | 1990-1994 | 0.65  (0.15, 1.14) | 1990-1993 | 0.45  (-0.14, 1.05) |
|  | 1994-2001 | -0.05  (-0.32, 0.21) | 1993-2001 | 0.23  (0.07, 0.39) |
|  | 2001-2005 | 0.05  (-0.73, 0.83) | 2001-2011 | -0.14  (-0.25, -0.03) |
|  | 2005-2010 | -0.29 (-0.79 - 0.20) | 2011-2015 | 0.07  (-0.51, 0.65) |
|  | 2010-2018 | -0.01 (-0.22 - 0.20) | 2015-2018 | 0.32  (-0.84, 1.48) |
|  | 2018-2021 | -0.28 (-1.05 - 0.50) | 2018-2021 | -0.25  (-0.83, 0.33) |
| **Japan** | 1990-1995 | -2.03  (-2.93, -1.12) | 1990-1994 | -1.35  (-1.83, -0.87) |
|  | 1995-2000 | 2.58  (1.27, 3.91) | 1994-2000 | 0.43  (0.09, 0.78) |
|  | 2000-2005 | -4.86  (-6.11, -3.61) | 2000-2005 | -1.13  (-1.61, -0.65) |
|  | 2005-2016 | 0.37  (0.03, 0.72) | 2005-2016 | 0.03  (-0.09, 0.15) |
|  | 2016-2021 | 4.09  (3.14, 5.06) | 2016-2021 | 1.45  (1.10, 1.79) |
| **Mongolia** | 1990-1997 | 0.21  (0.13, 0.29) | 1990-1995 | -0.05  (-0.13, 0.04) |
|  | 1997-2005 | 0.30  (0.22, 0.38) | 1995-2001 | 0.21  (0.12, - 0.29) |
|  | 2005-2010 | 1.41  (1.21, 1.60) | 2001-2010 | -0.20  (-0.24, -0.16) |
|  | 2010-2015 | -0.75  (-0.95, -0.56) | 2010-2015 | 1.63  (1.51, 1.75) |
|  | 2015-2021 | 0.35  (0.24, 0.45) | 2015-2021 | 0.06  (-0.01, 0.12) |
| **Republic of Korea** | 1990-1995 | -2.77  (-4.19, -1.32) | 1990-1994 | -1.87  (-2.45, -1.28) |
|  | 1995-1999 | 2.86  (-0.57, 6.41) | 1994-2000 | 0.39  (-0.03, 0.82) |
|  | 1999-2011 | -0.66  (-1.11, -0.21) | 2000-2005 | -1.20  (-1.79, -0.61) |
|  | 2011-2016 | -1.47  (-3.67, 0.78) | 2005-2016 | 0.00  (-0.15, 0.15) |
|  | 2016-2021 | 4.16  (2.59, 5.75) | 2016-2021 | 1.46  (1.03, 1.90) |
